# Enzyme-catalyzed synthesis of malonate polyesters and their use as metal chelating materials†

**DOI:** 10.1039/d1gc01783g

**Published:** 2021-06-29

**Authors:** Fergal P. Byrne, Jamie M. Z. Assemat, Amy E. Stanford, Thomas J. Farmer, James W. Comerford, Alessandro Pellis

**Affiliations:** Green Chemistry Centre of Excellence, Department of Chemistry, University of York Heslington York YO10 5DD UK alessandro.pellis@boku.ac.at fergal.byrne@york.ac.uk; SINTEF Forskningsveien 1A 0373 Oslo Norway; University of Natural Resources and Life Sciences, Vienna, Department of Agrobiotechnology, Institute of Environmental Biotechnology Konrad Lorenz Strasse 20 3430 Tulln an der Donau Austria

## Abstract

Following the environmental problems caused by non-degradable plastics there is a need to synthesise greener and more sustainable polymers. In this work we describe, for the first time, the facile enzyme-catalysed synthesis of linear polyesters using dimethyl malonate as the diester. These polymers, containing a different aliphatic diol component (C_4_, C_6_ or C_8_), were synthesised in solventless conditions using immobilized *Candida antarctica* lipase B as the biocatalyst. The potential of enzymes for catalysing this reaction is compared with the unsuccessful antimony- and titanium-catalysed synthesis (*T* > 150 °C). The application of the synthesized polymers as effective metal chelators in biphasic, green solvent systems was also described, together with the characterisation of the synthesised materials.

## Abstract 2

Following the environmental problems caused by non-degradable plastics there is a need to synthesise greener and more sustainable polymers.

## Graphical abstract

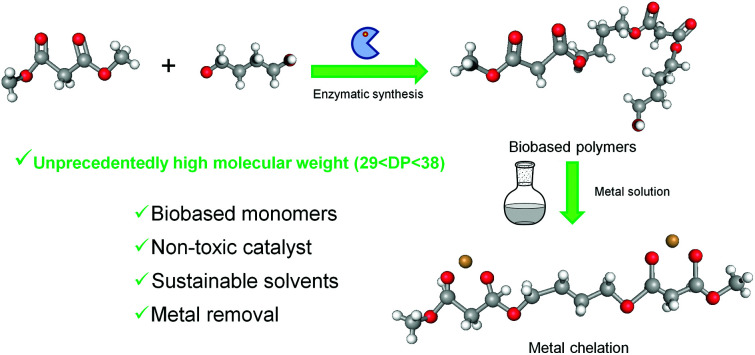

## Introduction

Due to the functionality limitations and the pollution caused by fossil-derived polymers,^1^ the chemical industry urges the development of greener routes to produce novel bio-based, degradable (or compostable) materials. Several steps in this direction were recently made, especially in the polyesters field, with the development of poly(ethylene 2,5-furandicarboxylate) (PEF) as a poly(ethylene terephthalate) (PET) substitute due to its similar mechanical and barrier properties^2,3^ and better biodegradability.^4,5^ Other furan- and pyridine-based polymers potentially useful for packaging and film applications were developed, but their synthesis remains limited to laboratory-scale.^6^

Recently, the potential of enzymes as green and selective biocatalysts has been demonstrated on several aromatic and aliphatic monomers, which is of great interest when the polycondensation of monomers carrying lateral functionalities is desired, as highlighted in several review articles.^7,8^ Such functionalities – the vinyl group of itaconic acid, the secondary hydroxy group of glycerol, sorbitol or mucic acid, *etc*. – are prone to a wide array of side reactions (such as Ordelt saturation, radical crosslinking, *etc*.) when traditional metal- or acid-catalyzed polymerization reactions are carried out.^9,10^ In fact, previously synthesized metal-chelating polymers based on a diethylenetriaminepentaacetic acid pendant group required a Michael addition of a thiol to be carried out in a second reaction step, and the double bond of the itaconate moiety was preserved only thanks to the use of very toxic chemicals such as 2-furanmethanethiol and a phosphazene base as the initiator.^11^

In this work, attention was focused on malonate-derived aliphatic polyesters. Malonic acid is a source of bio-based 1,3-diketone functionality, and it is produced commercially by Lygos using engineered yeast strains.^12^ To the best of our knowledge, malonate-derived polyesters were reported before in the literature only in the form of short oligomers having a maximum DP of around 5.^13^ In fact, the reports dealing with malonate polymers describe mainly the synthesis of aliphatic hyperbranched polyesters (HBPE) from various monomers derived in one step from commercial diethyl malonate.^14^ The acid- and metal-catalyzed polycondensation of malonate derivatives bearing aliphatic residues^15^ was also reported together with the sequential anionic polymerization of ethylene oxide and methylidene malonate to obtain poly(ethylene oxide)-*block*-poly(methylidene malonate 2.1.2) block copolymers bearing a primary amino group at the PEO chain end.^16^ The only work where the malonate unit was changed from a malonic acid or dialkyl ester to being part of the main chain, is the work of Doğan and Küsefoğlu, published in 2008, that reported the 1,4-diazabicyclo[2.2.2]octane-catalyzed synthesis of a biodegradable polymeric foam from epoxidized soybean oil and malonic acid.^17^

There has been increased interest in elemental sustainability in recent years. Commonly used metals such as cobalt, nickel, copper and zinc have reserves expected to last only 50–100 years. Recovery of these metals from waste streams is vital to maintain supplies of these dwindling resources.^18^ One method of recovering metals from aqueous waste streams is solvent-based hydrometallurgy.^19,20^ This involved contacting an organic solution containing metal chelators with an aqueous metal solution. A biphasic system emerges, in which metal ions can pass from the aqueous phase to the metal chelators in the organic phase. The metals can be recovered from the organic phase by re-extraction by an acidic solution, allowing the free chelators in the organic phase to be reused.^20^ However, issues of toxicity, bioaccumulation and persistence in the environment of chelators are common.^21^ In addition, most currently available chelators are petroleum-derived with few examples of bio-based products (Nouryon Dissolvine range being a rare example).^22^ As such, bio-based, safe, water-insoluble metal chelators are sought after.

**Fig. 1:**
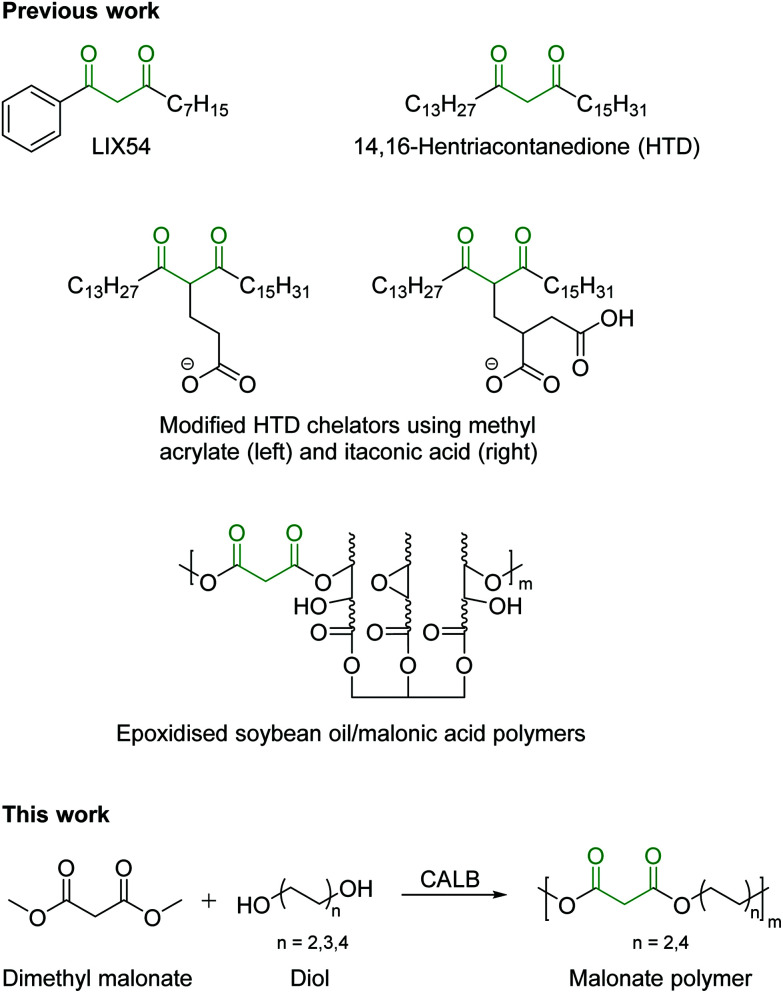
Structures of the previously described 1,3-diketone chelators LIX54, 14,16-hentriacontanedione and the malonate-based polymers described in this work.

Many functional groups can be used as chelators, such as oximes, carboxylates, phosphorous acids, and 1,3-diketones. 1,3-Diketones such as LIX54 (Fig. 1) are commercially available for this purpose,^19,23^ but naturally occurring other 1,3-diketones such as 14,16-hentriacontanedione are present in plant waxes.^24,25^ Indeed, biphasic extraction systems have been proposed in the past using bio-derived lipophilic chelators sourced from wheat straw wax,^25^ as well as modified wax products to produce super-chelators.^24^

Herein we present the facile enzyme-catalyzed synthesis of dimethyl malonate-based linear polyesters having a different aliphatic diol component (C_4_, C_6_ or C_8_). The reaction was conducted in solventless conditions using immobilized *Candida antarctica* lipase B as the biocatalyst. The potential of enzymes for catalysing this reaction is compared with the largely unsuccessful chemo-catalytic metal-catalyzed synthesis. The application of the synthesized polymers as effective metal chelators in biphasic, solvent-based hydrometallurgy is also described together with a detail characterization of the synthesized materials. Moreover, these aliphatic polyesters are known to be easily degraded to their constituent monomers (diacids and polyols) using a variety of hydrolytic enzymes (*e.g.* lipases, cutinases),^26,27^ therefore allowing the recovery of such building blocks and the re-synthesis of the polymer in a closed-loop circular economy concept.

### Synthesis of malonate-based aliphatic polymers

Quite surprisingly, very few reports describing the use of malonic acid (or its esters) as the diacid component of polyesters were found in the literature^28,29^ and none of them focuses on the chelating properties of these polymers. We therefore initially attempted to synthesize malonate polyesters using the most commonly known metal catalysts for polycondensation reactions: antimony oxide and titanium butoxide, catalysts widely known for the synthesis of PET,^30^ PEF^31^ and a wide range of other aliphatic and aromatic polyesters.^32^ Unfortunately, when using dimethyl malonate (DMM) as the diester in combination with various aliphatic polyols, the metal-catalyzed synthesis was unsuccessful, with obtained *M*_n_s between 1000 and 2600 Da (DP of 6 and 16 respectively, Table 1).

**Table 1:** Metal-catalyzed synthesis of malonate polyesters

Diol	Catalyst	*M*_n _ a [Da]	*M*_w _ a [Da]	*Đ* a
1,4-BDO	Sb_2_O_3_	2100	4000	1.90
	Ti(O^*t*^Bu)_4_	2600	4600	1.79
	Sb_2_O_3_	1000	2400	2.28
1,8-ODO		1600	2800	1.68

a Calculated *via* GPC.

The rather low molecular weights obtained in this work using metal catalysts can be explained with the fact that β-diketones, such as malonates, are known chelating agents and can therefore competitively chelate the catalyst metal ions, reducing their capacity to promote the transesterification reaction. This idea is supported by the fact that titanium forms complexes with dimethyl malonate^33^ creating a useful catalyst for the polymerization of polypropylene.^34^ One of the few available reports on malonate polyesters is the P_2_O_5_-catalyzed synthesis of poly(1,3-propyl malonate), but also in this case the obtained molecular weights were really limited since the maximum achieved DP was approximately 5.^13^

Taking inspiration from recent papers on environmentally friendly synthesis of polymers, and due to the impossibility to obtain polymers using traditionally-used methods, an enzymatic approach was used in order to synthesize a series of malonate-containing aliphatic polyesters using diols having a chain length from 4 to 8 carbon atoms. For the synthesis performed in this work, an immobilized preparation of *Candida antarctica* lipase B (iCaLB) was used as the biocatalyst since this enzyme was reported to be an excellent candidate for such synthesis reactions.^7,35^ The polycondensation reaction progressed at very mild (85 °C, 1000/20 mbar, 6 + 18 h) solventless conditions. The application of such environmentally-friendly synthesis protocol was possible since DMM is a liquid at the used operational temperature while the corresponding diacid, malonic acid, has a melting temperature reported to be between 135 °C and 137 °C.

**Fig. 2:**
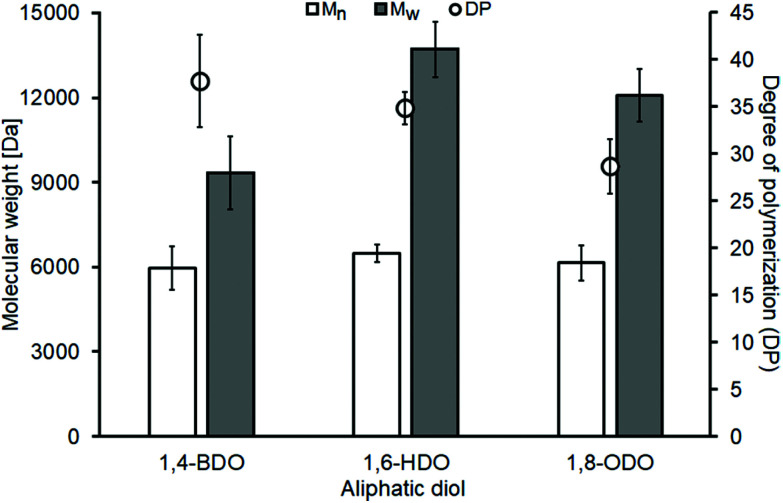
Enzymatic synthesis of linear malonate-based polyesters. Number average molecular weights (*M*_n_, white bars) and weight average molecular weights (*M*_w_, grey bars) were determined *via* gel permeation chromatography using polystyrene standards. The degree of polymerization (DP, white circles) was determined dividing the *M*_n_ by the *M*_0_ (weight of the repetitive unit of the polymer). All experiments were performed in duplicates and shown ± the standard deviation.

The enzymatic synthesis experiments show similar *M*_n_ values of around 6000 Da for all the used diols while the *M*_w_ values increase from 9000 Da to 12–14 K Da with the increase of the diol's chain length (Fig. 1). The reported values are in line with previous reports of solvent-free enzymatic polycondensations where the used diester was dimethyl adipate that also showed similar *M*_n_ (∼7000 Da) and increasing *M*_w_ (from 11 to 14 K Da) when the same three aliphatic diols having increasing carbon chain length were used.^35^ The decrease of the DP as the diol increases, as seen for both the malonate and the adipate polyesters, is also a common trait (Fig. 2).^7,35^

All synthesized malonate-based polyesters have 29 < DP < 38, therefore presenting molecular masses significantly higher in comparison of the short oligomers previously synthesized using P_2_O_5_ and our own chemocatalytic synthesis approach (Table 1). The polymers were recovered using a simple vacuum filtration that allowed the removal of the immobilized enzyme and the work-up solvent was then removed *via* rotary evaporation. All isolated polyesters were colourless viscous liquids and subsequently used for the chelation experiments without further purification.

Polymer structures and relative monomer conversions were elucidated *via*^1^H-NMR spectroscopy (Fig. S1–S5 in ESI†). Upon reaction, the –CH_2_–CH̲_2_–OH signal from the diol has a characteristic change of chemical shift from 3.65 ppm to 4.14 ppm, proving the formation of an ester bond. Additionally, the –OCH̲_3_ signal from the malonate, observable at 3.75 ppm disappears due to the release of MeOH with the progression of the reaction. The signals at 3.38 ppm (C–CH̲_2_–C of the malonate) and in the 1.3–1.8 ppm range (–CH̲_2_–CH_2_–OH of the diols) do not noticeably change chemical shift upon elongation of the polymer chain. ^13^C-NMR spectroscopy of the polymer reveals the typical signals for these aliphatic polyesters (Fig. S6 in ESI†).

### Metal chelation in biphasic systems based on green solvents

The malonate polyesters were assessed for their ability to extract metal ions from aqueous streams in a biphasic system. The biphasic system involved dissolving the polyester in an organic solvent and mixing it with a metal-containing aqueous solution. Upon contact between the two phases, metal transfer occurs from the aqueous phase to the diketone chelating points on the polyester in the organic phase, purifying the aqueous phase. Separation of the two phases and re-extraction of the organic phase with an acidic stripping solution can recover the metal from the polyester for reuse, and also regenerate the diketone chelating point on the polyester.

Copper was chosen as the target metal for chelation using the polyesters due to it being a common pollutant in metallurgy waste streams.^36^ Cl^−^ was selected as the counter ion as it has previously been shown to be effective in the chelation of copper using diketone species due to it being a strong inner-sphere ligand.^24,25^ The chelation tests were carried out across as pH range of 8.4–12.3.

Several requirements exist for the choice of organic solvent for this purpose. It must partition well with water; the chelating agent must favour solubility in the organic solvent over water; it must facilitate enol formation; and it must not be reactive in the extraction conditions. As the solvent does not need to be evaporated at any stage of the extraction process, a low boiling point is not necessary. In fact, a higher boiling point will reduce losses to the atmosphere, improving the economics of such an extraction process. In addition, it will add to the green credentials of the process, as emissions, exposure to workers and solvent demand are reduced. The CHEM21 solvent selection guide recommends a boiling point of between 70–139 °C.^37^

Three candidate solvents were ultimately selected using the CHEM21 solvent guide:^37^*para*-cymene, ethyl levulinate and anisole. *para*-Cymene and anisole have boiling points within the ideal range (70–139 °C), while that of ethyl levulinate is 206 °C. However, as the solvent/polymer mixture can be used repeatedly, this higher than preferred boiling point is not a significant issue. All three candidates are aprotic, meaning the enol form is more likely to be favoured^25^ which is a prerequisite for chelation ability (keto form is stabilised by intermolecular hydrogen-bonding in protic solvents).^38^ Polymer insolubility in BDO MAL polymer prevented *para*-cymene from being tested, while ethyl levulinate likely chelated with metal ions during extraction, indicated by a green complex being formed when mixed with copper solutions. Anisole could dissolve all polymers, and also scores well in the CHEM21 solvent selection guide, being classified as “recommended”.^37^ As such, anisole was chosen as the most appropriate solvent for this process.

**Fig. 3:**
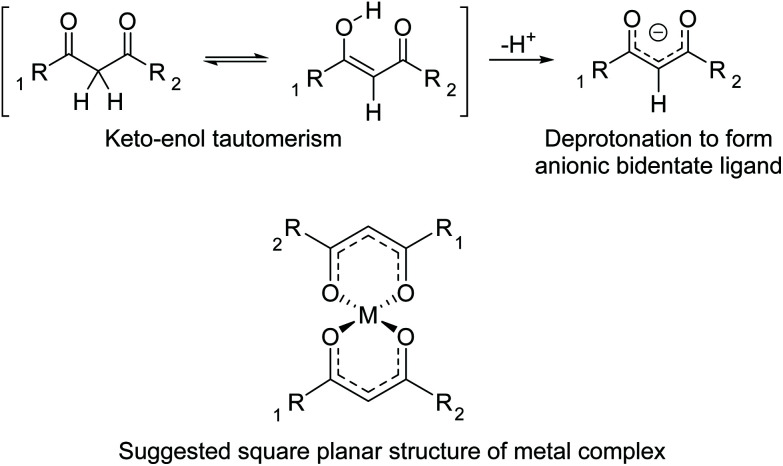
Keto–enol tautomerism of 1,3-diketones followed by deprotonation to form negatively charged bidentate ligand.

The 1,3-diketones functionality can exist in keto and enol forms (Fig. 3). In basic conditions, deprotonation of the acidic proton in the 2-position forms an anionic bidentate ligand which are weak chelators. A square planar complex is suggested, similar to that of bis(acetylacetonato)copper(ii), as previously determined by single crystal X-ray diffraction.^39^ As the polymers existed as a viscous liquid, possibly due to residual solvent that could not be removed *in vacuo*, powder XRD could not be carried out to confirm the square planar complex in the malonate polymers of this work.

The polymers with the highest (ODO MAL) and lowest (BDO MAL) *M*_0_ were chosen as model to perform chelation tests with CuCl_2_. A pH range of 8.4–12.3 was used for the tests. Chelation tests were carried out in a biphasic system consisting of a CuCl_2_-rich aqueous phase (0.05 M CuCl_2_, 0.25 M NH_3_) and an organic solution of the chelating polymer (0.05 M). Interestingly, the density of the aqueous metal solutions at higher pH (10.4–12.3) changed such that the layers switched in the biphasic system when the BDO MAL was used. This is due to the proximity of the density of pure anisole (0.995 g mL^−1^) to that of water (1.000 g mL^−1^), which must be taken into consideration in an industrial process.

**Fig. 4:**
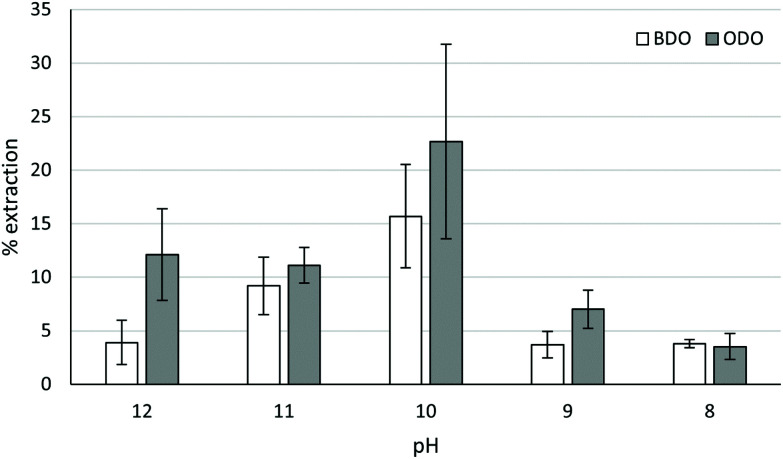
The % extraction of Cu(ii) from ammoniacal solutions using “BDO” MAL and “ODO” MAL at different pHs and 1 : 1 by weight loading of chelator : Cu(ii). The pH shown in the graph has been rounded to the nearest whole number for visual clarity. Exact pH values are shown in the ESI.†

Fig. 4 shows that optimal pH for extraction was similar in both cases, with ∼pH 10 being optimal for ODO MAL and BDO MAL polymers (specific pH's and absorbances for chelation tests are shown in Tables S2 and S3 in ESI†). This is consistent with previous observations of 1,3-diketones being more effective in basic conditions.^17^ Superior extraction was obtained with the ODO MAL, with mean Cu extraction of 22.7% compared to 15.7% for the BDO MAL polymer. Extraction with the malonate polymers is comparable with the commercial LIX54 (18% in a 50 : 50 mix of LIX54/kerosene, 40% using pure LIX54),^23^ demonstrating the potential of such a chelating polymer for use in an industrial setting. Further work is required on optimising the conditions for extraction (polymer loading, further solvent investigation, more robust pH control), their stability over multiple uses and their affinities for other metals in mixed aqueous streams which will be the focus of a subsequent full article.

## Conclusions

A series of malonate polymers were successfully synthesized using environmentally-friendly conditions (enzymatic catalyst, *T* < 90 °C, solvent for the workup: MeTHF) and used to achieve the efficient chelation of copper, a common pollutant in metallurgy waste streams. A superior extraction efficiency of 23% was obtained with the ODO MAL polymer that is comparable with commercially-available LIX54 (18% in a 50 : 50 mix of LIX54/kerosene, 40% using pure LIX54), demonstrating the potential of this new class of polyesters for use in an industrial setting. Enzymatic catalysis, showing high selectivity, low operational temperatures and benign reaction conditions, is emerging as a useful tool to complement chemo-catalytic routes for the synthesis of multifunctional polymers having structures that are otherwise not possible to obtain using traditional metal- and acid based-methods.

## Author contributions

A. P., J. M. Z. A. and A. E. S. performed the enzymatic polymer synthesis and material's characterization. F. P. B., J. M. Z. A. and A. E. S. performed the chelation experiments. A. P. and J. W. C. performed the chemo-catalytic synthesis. A. P. and F. P. B. planned the experiments and wrote the manuscript. F. P. B., A. P. and T. J. F. supervised the work. All authors corrected the manuscript and discussed the data prior to submission.

## Conflicts of interest

The authors declare no conflict of interests.

## Supplementary Material

GC-023-D1GC01783G-s001
