# Supplementary material for: Enzyme-catalyzed synthesis of malonate polyesters and their use as metal chelating materials
Source: Green Chem. 2021 Jun 29;23(14):5043–8. doi: 10.1039/d1gc01783g (PMC8293702; doi:10.1039/d1gc01783g)
Supplement: GC-023-D1GC01783G-s001 [file GC-023-D1GC01783G-s001.pdf]

Electronic Supplementary Material (ESI) for Green Chemistry.
This journal is © The Royal Society of Chemistry 2021

Electronic Supplementary Information

Enzyme-catalyzed synthesis of malonate polyesters and their use as metal chelating materials

Fergal P. Byrne[1,*], Jamie M. Z. Assemat[1], Amy E. Stanford[1], Thomas J. Farmer[1], James W. Comerford[1, 2], Alessandro Pellis[1, 3, *]

[1]. University of York, Department of Chemistry, Green Chemistry Centre of Excellence, YO10 5DD, Heslington, York, UK

[2] SINTEF, Forskningsveien 1A, 0373, Oslo, Norway

[3] University of Natural Resources and Life Sciences, Vienna, Department of Agrobiotechnology, Institute of Environmental Biotechnology, Konrad Lorenz Strasse 20, 3430, Tulln an der Donau, Austria

* Correspondence to: Dr. Alessandro Pellis, alessandro.pellis@boku.ac.at and Dr. Fergal Byrne, fergal.byrne@york.ac.uk

Materials and methods

Chemicals and enzymes

All chemicals, monomers and enzymes were purchased from Sigma-Aldrich and used as received if not otherwise specified. *Candida antarctica* lipase B immobilized on a macroporous acrylic resin was purchased from Sigma-Aldrich (product code: L4777).

Chemocatalytic synthesis

38 mmols (5.01 g) of dimethyl malonate was added to a flask along with 47.5 mmols (4.28 g) 1,4-butanediol and 1 mol% of the metal catalyst and a magnetic stirrer bar. A short path distillation arm and condenser was connected and the whole system was evacuated to 1 mbar and backfilled with argon four times. The flask was then heated to 150 °C under argon, stirring at 300 rpm. After 5 hours the temperature was increased to 180 °C for 1 hour followed by application of 200 mbar vacuum overnight. After 17 hours the flask was removed from the heat and back filled with argon before

analysis. The polymers appearances were a fluid white paste or a sticky yellowish liquid when Sb_2O_3 or Ti(iv) tetra*tert*-butoxide were used as the catalyst, respectively.

Biocatalyzed synthesis

0.006 mol of diester and 0.006 mol of diol (diester:diol ratio= 1.0:1.0) were added in a 25-mL round bottom flask. The mixture was then stirred at 85 ºC until a homogeneous melt was obtained. 10% w w^{-1} (calculated on the total amount of the monomers) of iCaLB was then added and the reaction was run for 6 h at 1000 mbar. A vacuum of 20 mbar was subsequently applied for additional 18 h maintaining the reaction T at 85 ºC. The reaction products were dissolved in MeTHF and the biocatalyst filtered off. The solvent was then removed via rotary evaporation and the products mixture analyzed without carrying our any additional purification steps.

Chelation experiments

Equal volumes (0.75 mL) of polymer-containing organic solution (0.05 M) and an aqueous metal solution (0.25 M NH_3, 0.05 M metal salt) were added to a vial. The pH of the aqueous solution was controlled by the addition of HCl. The mixture was shaken and allowed to stand for at least 1 hour (no change was observed between standing for 1 hour or overnight), before centrifuging (3,500 rpm, 4 minutes, 20 °C). Aliquots of 0.2 mL or 0.4 mL were taken from the aqueous layer for analysis by UV-vis. spectroscopy. Dilutions were made as necessary prior to analysis.

Nuclear Magnetic Resonance (NMR)

^{1}H-NMR spectroscopy was performed on a JEOL JNM-ECS400A spectrometer at a frequency of 400 MHz. $CDCl_3$ was used as the NMR solvent for all synthesized polymers.

Gel Permeation Chromatography (GPC)

Polymeric samples were diluted in THF at a concentration of ~2 mg mL^{-1}. ~5 µL of toluene were added in each sample as internal standard. GPC runs were carried out using a PSS SDV High set composed of 3 analytical columns (300 × 8 mm, particle diameter 5 µm) of 1000, 105 and 106 Å pore sizes with a PSS guard column (Polymer Standards Service GmbH) installed in a PSS SECcurity SEC system. Elution was

carried out using THF as the mobile phase (1 mL min^{-1}). Column temperature was maintained at 23 ºC and detection was performed by refractive index detector. Elution time was of 50 min. Calibration was carried out in the molecular weight range 370-2520000 Da using the ReadyCal polystyrene standards supplied by Sigma Aldrich and referenced to the toluene peak.

Supplementary Figures

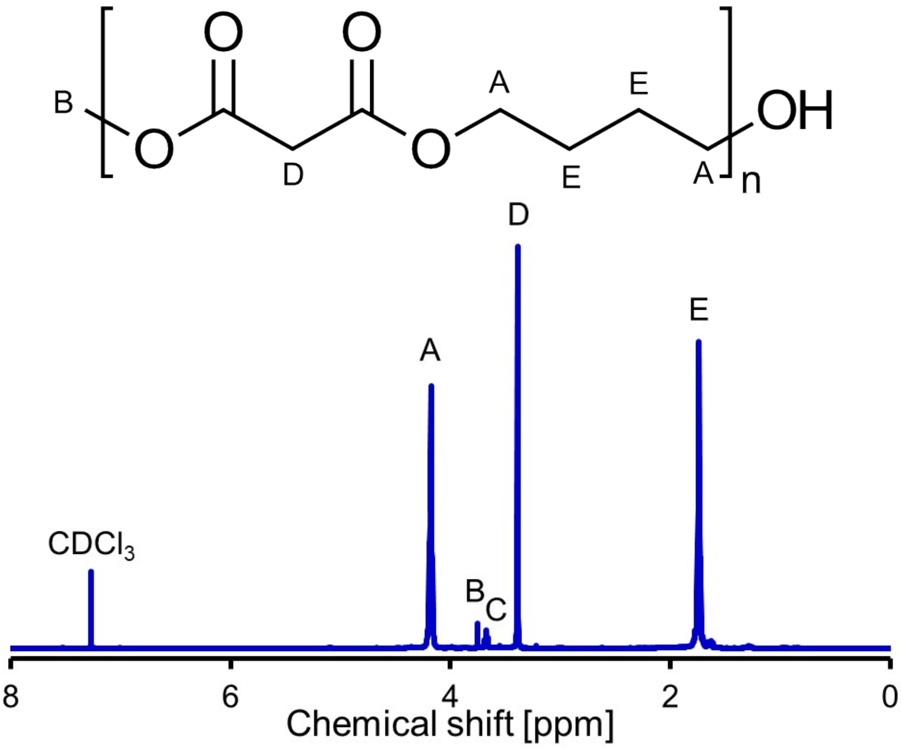

Figure S1. ^{1}H-NMR spectrum of the reaction between dimethyl malonate (DMM) and 1,4-butanediol (BDO) catalyzed by iCaLB. B: unreacted or terminal -OC<u>H</u>$_3$ groups from DMM; C: unreacted or terminal CH_2-C<u>H</u>$_2$-OH from BDO.

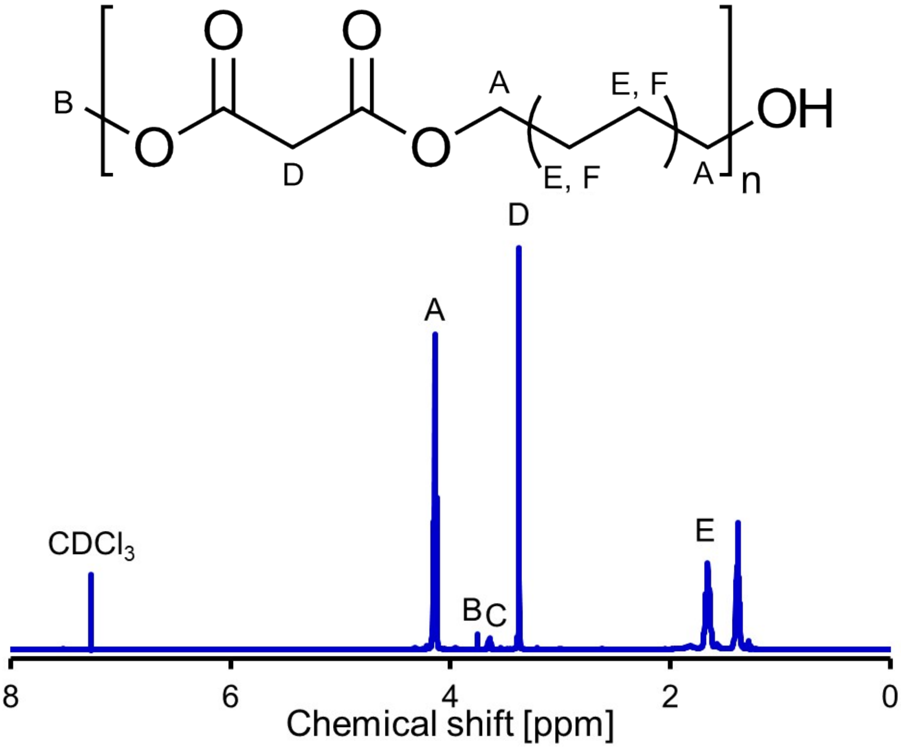

Figure S2. ^{1}H-NMR spectrum of the reaction between dimethyl malonate (DMM) and 1,6-hexanediol (HDO) catalyzed by iCaLB. B: unreacted or terminal -OC<u>H</u>$_3$ groups from DMM; C: unreacted or terminal CH_2-C<u>H</u>$_2$-OH from HDO.

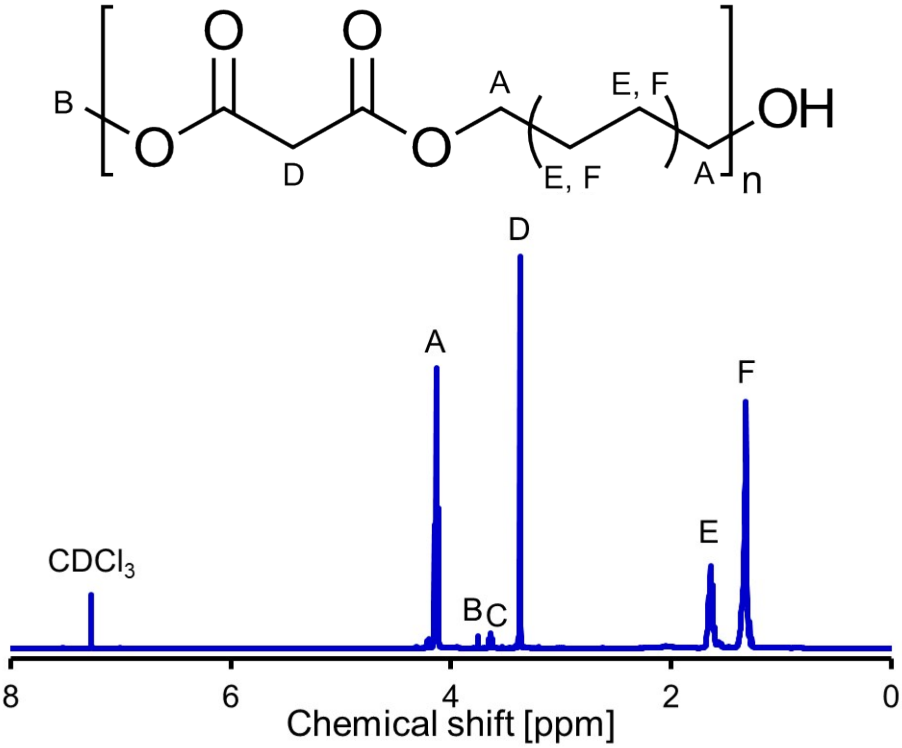

Figure S3. ^{1}H-NMR spectrum of the reaction between dimethyl malonate (DMM) and 1,8-octanediol (ODO) catalyzed by iCaLB. B: unreacted or terminal -OC<u>H</u>$_3$ groups from DMM; C: unreacted or terminal CH_2-C<u>H</u>$_2$-OH from ODO.

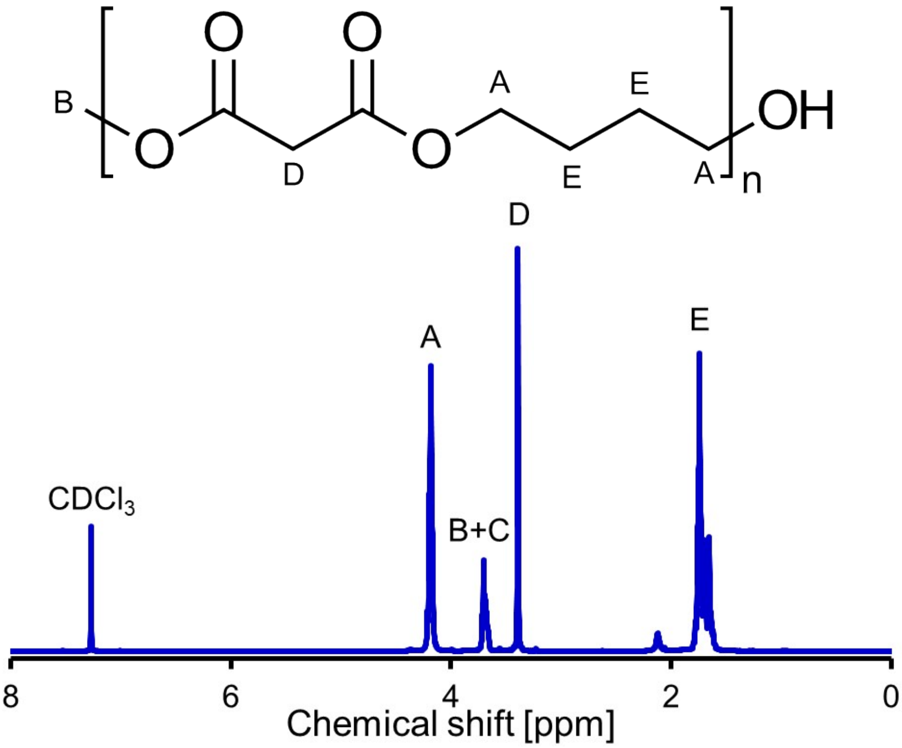

Figure S4. ^{1}H-NMR spectrum of the reaction between dimethyl malonate (DMM) and 1,4-butanediol (BDO) catalyzed by Sb_2O_3. B: unreacted or terminal -OC<u>H</u>$_3$ groups from DMM; C: unreacted or terminal CH_2-C<u>H</u>$_2$-OH from BDO.

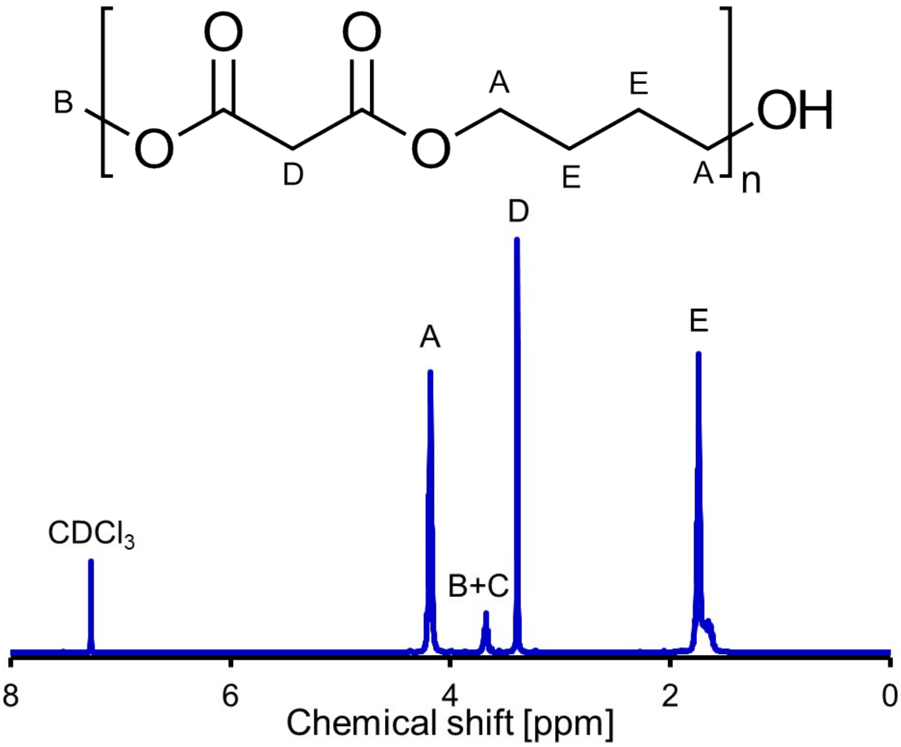

Figure S5. ^{1}H-NMR spectrum of the reaction between dimethyl malonate (DMM) and 1,4-butanediol (BDO) catalyzed by $Ti(OBu)_4$. B: unreacted or terminal -OC$\underline{H_3}$ groups from DMM; C: unreacted or terminal CH_2-C$\underline{H_2}$-OH from BDO.

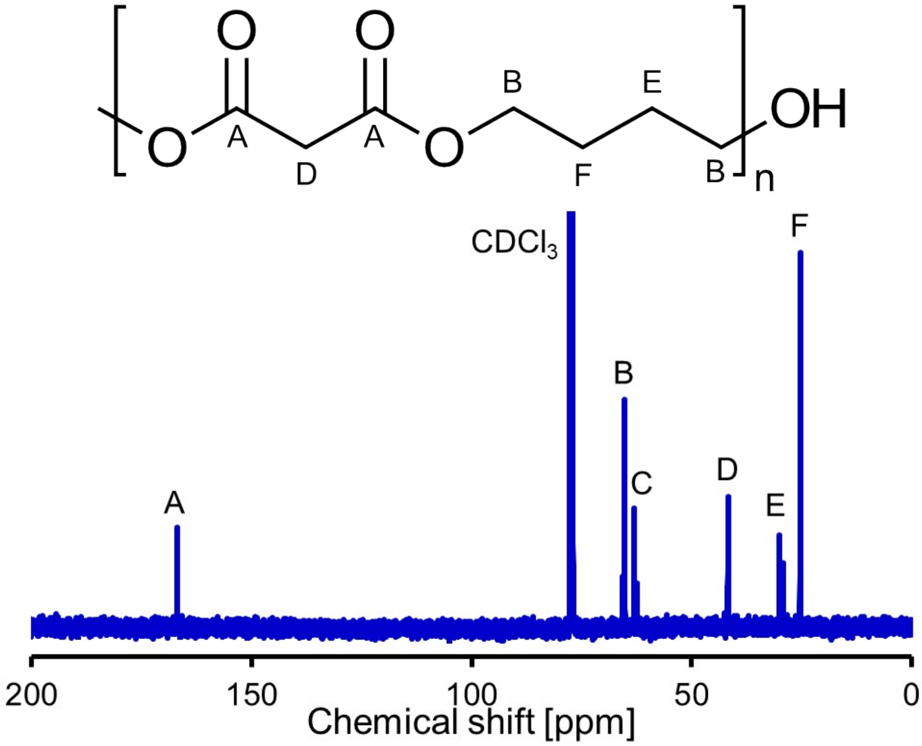

Figure S6. ^{13}C-NMR spectrum of the reaction between dimethyl malonate (DMM) and 1,4-butanediol (BDO) catalyzed by Sb_2O_3.

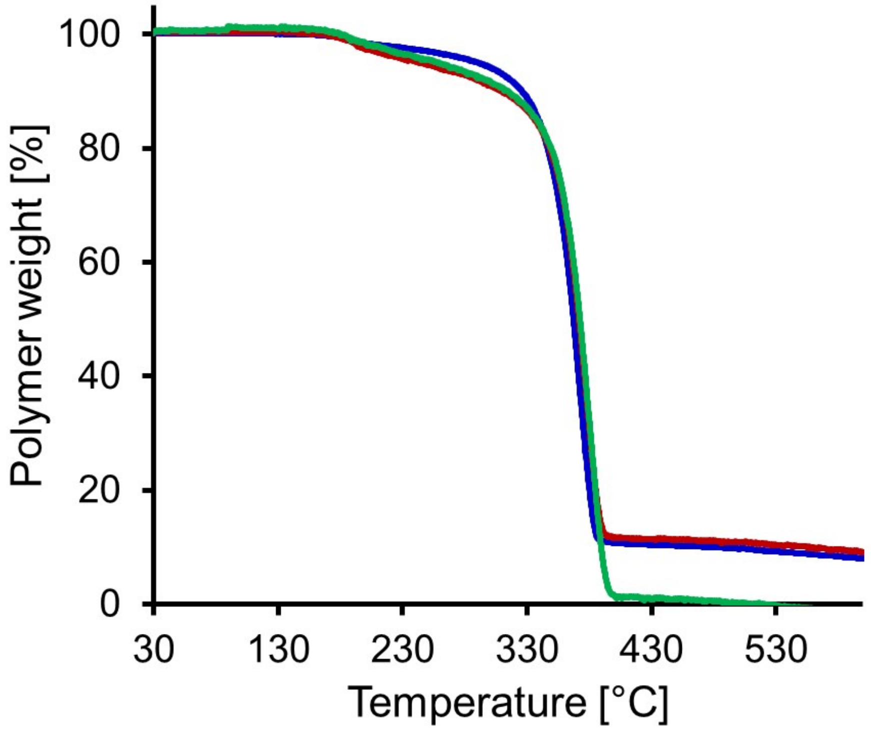

Figure S7. Thermogravimetric analysis of the enzymatically synthesized polyesters. The Td_{50} values based on the different diols' chain length were: BDO (blue): 367 °C, HDO (green): 372°C, ODO (red): 373°C.

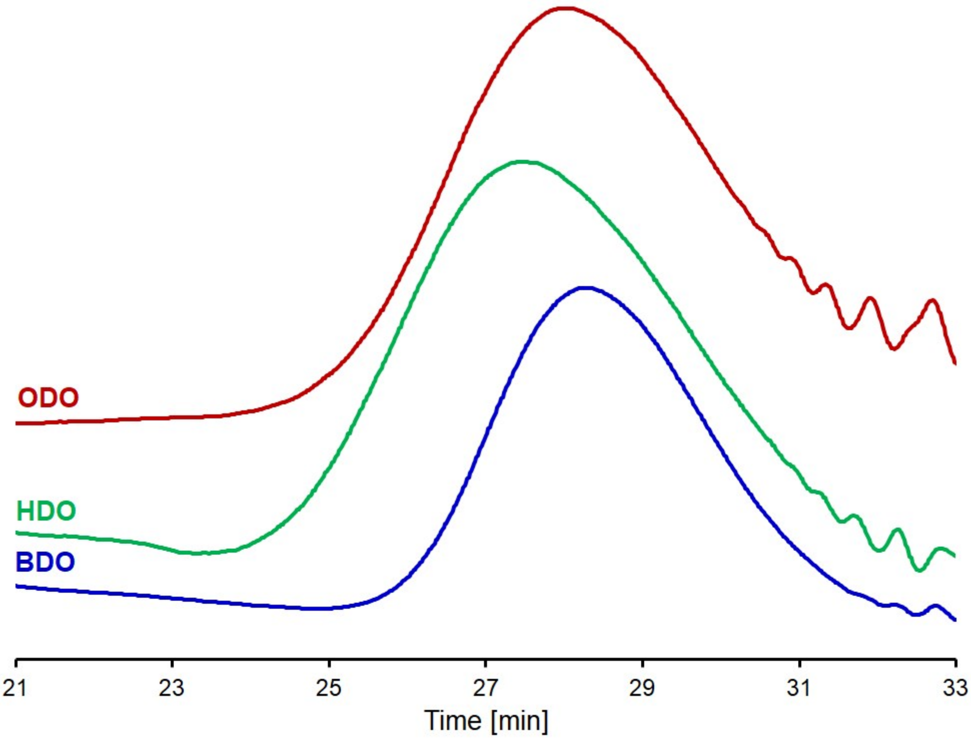

Figure S8. Gel permeation chromatography of the enzymatically synthesized polymers using BDO (blue), HDO (green) and ODO (red).

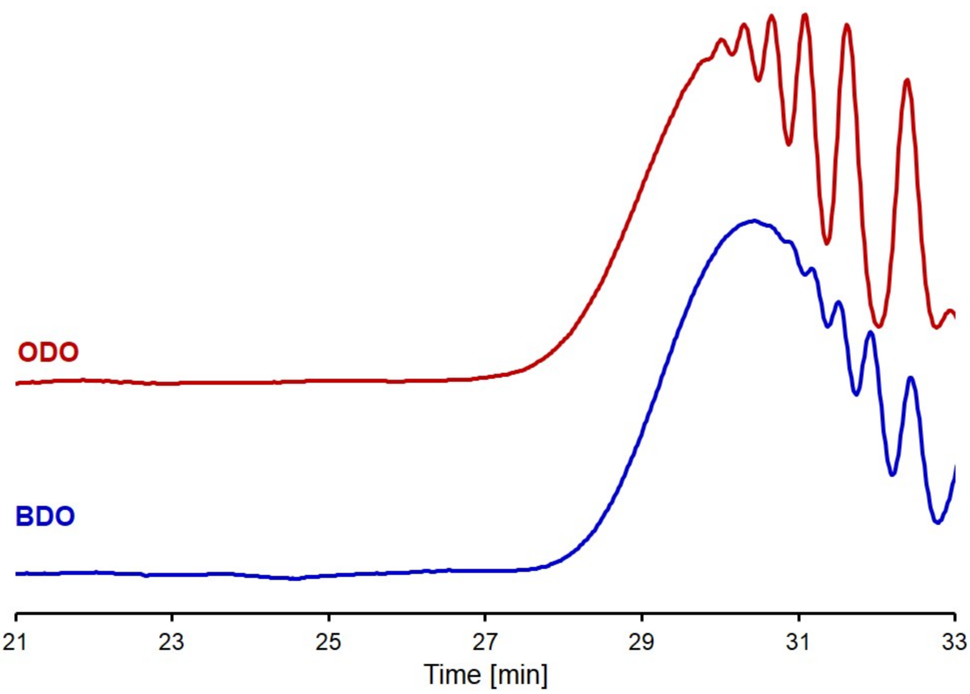

Figure S9. Gel permeation chromatography of the Sb_2O_3 synthesized polymers using BDO (blue) and ODO (red).

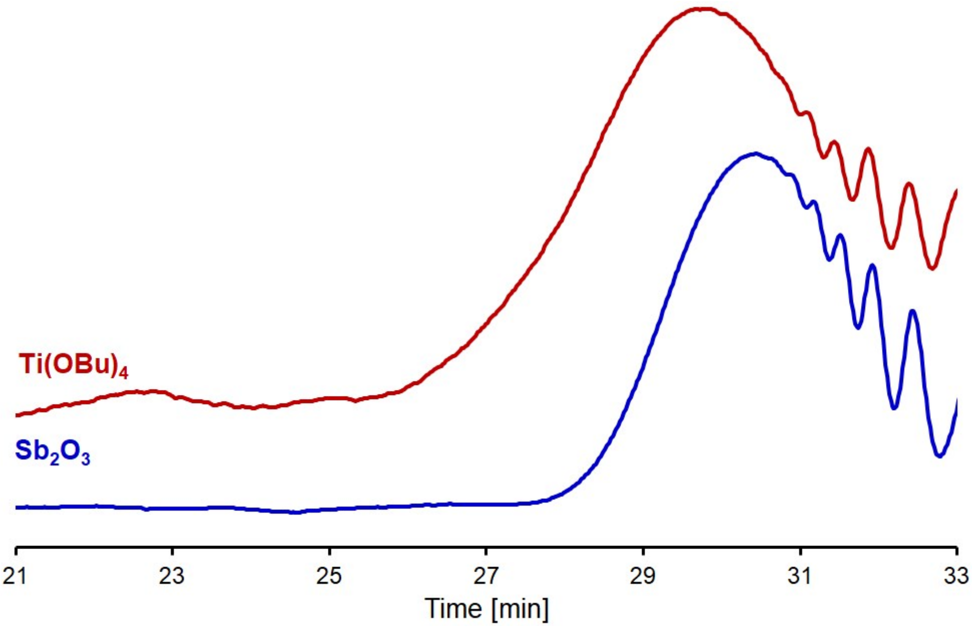

Figure S10. Gel permeation chromatography of the Sb_2O_3 (blue) and $Ti(OBu)_4$ (red) synthesized polymers using BDO as the diol.

Supplementary Tables

Table S1. Summary of the gel permeation chromatography analysis performed on all synthesized malonate-based polymers.

Diol	Catalyst	GPC		
		M_n [Da]	M_w [Da]	Đ
1,4-BDO	Sb_2O_3	2100	4000	1.90
	$Ti(OBu)_4$	2600	4600	1.79
	Sb_2O_3	1000	2400	2.28
1,8-ODO		1600	2800	1.68
1,4-BDO	iCaLB	5400	8400	1.55
		6500	10200	2.00
1,6-HDO		6700	13000	1.94
		6300	14400	2.30
1,8-ODO		6600	12700	1.93
		5700	11400	2.28

Table S2. Chelation data for the BDO MAL polymer.

HCl / M	pH	Abs. / %	λ_{max} / nm	ε / dm^3 mol^{-1} cm^{-1}	[$CuCl_2$] / M	Initial [$CuCl_2$] / M	Extr. / %	s	M	μ
0.00	12.25	1.199	622	55.49	0.0432	0.0461	6.3	2.1	3.0	3.9
	12.25	1.242	619	55.49	0.0448	0.0461	3.0			
	12.25	1.248	621	55.49	0.0450	0.0461	2.5			
0.25	10.98	1.206	616	56.04	0.0430	0.0486	11.5	2.7	9.8	9.2
	10.98	1.230	616	56.04	0.0439	0.0486	9.8			
	10.98	1.278	616	56.04	0.0456	0.0486	6.3			
0.75	10.36	1.212	615	56.65	0.0428	0.0504	15.1	4.8	15.1	15.7
	10.36	1.131	614	56.65	0.0400	0.0504	20.8			
	10.36	1.269	615	56.65	0.0448	0.0504	11.2			
2.00	9.22	1.307	598	54.72	0.0478	0.0491	2.7			
	9.22	1.275	598	54.72	0.0466	0.0491	5.1			
	9.22	1.297	598	54.72	0.0474	0.0491	3.4			
2.54	8.43	1.275	598	52.58	0.0485	0.0503	3.6	0.4	3.6	3.8
	8.43	1.266	597	52.58	0.0481	0.0503	4.2			
	8.43	1.274	597	52.58	0.0485	0.0503	3.6			

Table S3. Chelation data for the ODO MAL polymer.

HCl / M	pH	Abs. / %	λ_{max} / nm	ε / dm^3 mol^{-1} cm^{-1}	[$CuCl_2$] / M	Initial [$CuCl_2$] / M	Extr. / %	s	M	μ
0.00	12.25	0.594	610	55.49	0.0428	0.0461	7.1	4.3	14.5	12.1
	12.25	0.592	609	55.49	0.0427	0.0461	14.7			
	12.25	0.593	610	55.49	0.0427	0.0461	14.5			
0.40	10.82	0.602	607	55.82	0.0431	0.0479	9.9	1.7	11.1	11.1
	10.82	0.612	606	55.82	0.0439	0.0479	12.3			
1.00	10.25	1.184	612	56.54	0.0419	0.0488	16.2	9.1	22.7	22.7
	10.25	1.002	612	56.54	0.0355	0.0488	29.1			
2.00	9.22	1.255	598	54.72	0.0459	0.0491	8.3	1.8	7.0	7.0
	9.22	1.289	598	54.72	0.0471	0.0491	5.8			
2.54	8.43	1.267	598	52.58	0.0482	0.0503	4.2	1.2	4.2	3.5
	8.43	1.259	596	52.58	0.0479	0.0503	4.3			
	8.43	1.287	598	52.58	0.0489	0.0503	2.1			
